# Vital Actions Which Play an Important Part in Dental Caries

**Published:** 1873-11

**Authors:** Henry S. Chase

**Affiliations:** St. Louis.


### SELECTED ARTICLES.

### ARTICLE IV.

### *Vital Actions which Play an Important Part in Dental Caries.*

BY HENRY S. CHASE, M. D., D. D. S., ST. LOUIS.

The various theories in regard to the kind or kinds of action which constitute dental caries, probably have more or less of truth in them all. That the process which produces that disintegration of the dental tissues, which is usually called by the name of dental caries is wholly chemical or wholly vital I do not believe. The great number of observations which have been made in regard to the action which acids have in disintegration of the dental tissues, have well established the fact that the destruction of tooth substance is mainly produced by chemical decomposition.

I think that the greater number of the members of the dental profession believe that chemical decomposition is the *sole* process in the history of dental decay or dental caries. *Formerly* vital action had greater consideration in the production of this disease, than it has at the present time; and it is my object in this paper to present a few thoughts in favor of the vital movements which I believe undoubtedly play an important part in the process of dental disintegration, and to give to those vital movements a place in the history of this disease which I believe they deserve.

Many persons assume that all vital movements cease in the dentine and cementum when those tissues are fully formed. A still larger number declare that there is no vital action whatever in the enamel substance when *that* is fully formed. I believe that observation will establish facts that will prove their assumptions to be false. Admitted facts prove that vital processes are the sole cause of the production and completion of all the hard tissues of the teeth. And the history of the shedding of the temporary or milk teeth, proves that it is vital action alone which removes the roots and whole body of the dentinal crown. It is easily seen then that it is a living movement that builds up the tooth structure to its highest perfection; and also as patent that it is the same process, in a different direction, that takes down, particle by particle, or cell by cell, that same edifice which was so beautifully erected. Microscopical observations have proven that a barrier of defense is often interposed between a cavity of decay and the dental pulp, by an increased calcification of the dentine. Here is a vital action again; and this action takes place in the teeth of the middle aged, as well as in those of the young. Again, in cases of mechanical abrasion, where the teeth are worn down below the thickness of the enamel, we often find the surface of the dentine almost as hard and polished as the enamel itself; and this density of the dentine does not extend to a great depth, but on the contrary is generally limited to a thin shell of that portion of the dentine which is liable to the destructive abrasion; looking very much as though the tooth was endeavoring to protect itself by packing its tubuli with calciferous matter, and thereby forming a wall of defense.

In *spontaneous* abrasion, or that which is neither chemical or mechanical, may not the loss of substance be by *vital* action, or absorption? I think it very probable, as it would only be in harmony with the foregoing facts, and I believe that this heretofore obscure condition will be found, on further investigation, to be the *result* of vital absorption, whatever the exciting *cause* may be.

Some of the most careful observers declare that they have known, in their own experience, the absorption of dentine under a perfect plug to take place to the extent of exposing the dental pulp; the exposure taking place absolutely without dental decay, and to such an extent that there could be no mistake about it. A large number of intelligent men are quite sure, from a great number of observations, extending over many years of experience, that the permanent teeth of the adult may become less dense in the structure of the dentine than the same teeth were at a former period; in fact, an interstitial decalcification of that tissue having taken place. These facts prove, then, that the two opposite processes of calcification and decalcification, may and do take place in the perfected hard tissue of the teeth; and furthermore, that the basal structure itself, on the animal portion of the dentine, may be removed without chemical action.

Besides the foregoing facts, I wish to describe a condition of the enamel and dentine which I have very many times observed, namely: A black or brown spot is seen on the proximate surface of a molar or biscuspid tooth; it is smooth, hard and glossy; in fact, seems to have as dense a surface as glass, or as the uncolored and perfect enamel on the same tooth. A fine excavator, in passing over it, detects no abrasion or disintegration. On cutting into this discolored portion, the tissue appears as solid, for a little distance, as ordinary enamel. The *dentinal* surface of this enamel is somewhat lighter colored than the exterior. As soon as the *dentine* is reached we observe a softer condition, but the dentine is not discolored; still, it seems somewhat decalcified. On proceeding a little further we find this tissue still softer, apparently caused by a greater decalcification, and then we come again to normally hard dentine. In all this, there is evidently no loss of *basal* structure, either of enamel or dentine, but there is a loss of substance, and this is in the lime salts of the dentine. It is apparent that there has been no *external* loss of tooth substance.

There has been no chemical processes taking place, but there has been a vital movement in the interior of the tooth, in fact among the tubuli themselves, resulting in the process called absorption. The calcareous elements have been carried away through the interior vessels of the tooth, and not by a chemical wasting away from the exterior. A progressive history of this diseased condition, would result in showing that after a longer period, the exterior blackened surface would show signs of disintegration; the surface would become roughened, little pits would be found in it, and on cutting it with an excavator the enamel would be found softened and granular; it would easily break down under the instrument, and show discoloration as deep on its dentinal surface as on its exterior. Beneath the enamel the dentine would show a brownish discoloration, and an entire decalcification for a greater or lesser distance, until the normally hard and healthy dentine is reached. Now in this subsequent history of this disease, we probably have more or less chemical action to account for, and on *exterior* loss of substance produced by it; and this exterior chemical action of acids on the enamel will undoubtedly progress towards the interior or dentinal portion of the tooth, as rapidly as a fresh surface is presented by the washing or wasting away of the decomposed tissues.

Pertinent to the subject would be the question, what has caused the condition of things corresponding to the *earlier* history of the tooth described? I answer *necrosis* of the enamel was produced by pressure of a contiguous tooth, causing the circulation of the blood plasma to cease in the basal structure of the enamel, and discoloration taking place as a consequence of necrosis, as usual. The irritation or inflammation consequent on, or simultaneous with this necrotic process, caused the vital movement in the dentine which carried away its lime salts. It must be remembered that irritation of a tissue may produce, under apparently the same circumstances, either of two opposite vital movements: namely a loss of substance, or an increase of substance. This is known to be the case in the bones of the body, and in its softer tissues.

In the teeth themselves, exostosis is caused by the irritation of a dead and decomposing pulp, and at other times absorption of the cementum and dentine of the roots takes place from the same cause.

I have already instanced the absorption of dentine under a plug which had irritated the dental pulp by the thermal changes produced by the conducting powers of the metal. At other times the same irritation produced by the same cause, calls into activity those vital movements which protect the pulp by a new deposit of dental tissue between the pulp and the plug. And here we will call to mind again, that result of vital activity which interposes a denser wall of dentine around a carious cavity, in which chemical processes are in the active destruction of tooth substance.

With these facts before us, is it not probable that in many cases of chemical disintegration of the teeth, that the irritation produced in the dental tissues causes a vital movement resulting in rapid decalcification of the dentine, and perhaps, also absorption of the basal or animal substance of that tissue, instead of the opposite process of extra deposition of lime salts in the dentine as a barrier to the decaying process. Or, in other words, is it not probable that the irritation produced in a cavity of decay, by the decomposition of food, or other organized substances causes, in many cases, a *vital action* which results in *absorption* of the lime salts in near proximity to the scene of action, instead of the opposite process of super calcification.

It seems to me, from the foregoing observations, that, taking a broad view of the process of dental caries, we must give to vital action an important place in its history.

In conclusion I wish to say that I have read Dr. Chandler's translation of Leber and Rottenstein's "Researches on Dental Caries." These authors give great importance to the influence produced by the leptothrix buccalis which they say is almost invariably found in all carious dentine and enamel. This fungus is also found upon the tongue and upon the gums of persons who are not particular in cleansing the mouth daily. My conclusions, taking their *observations* as correct, which I believe they are, lead me to consider the leptothrix as an agent which cannot initiate dental caries, but as a concomitant of the disease may accelerate its progress. And I would here take occasion to heartily recommend the work of the authors on this subject to my professional brethren.—*Missouri Dental Journal.*

